# ﻿Complete mitochondrial genome sequences of *Physogyralichtensteini* (Milne Edwards & Haime, 1851) and *Plerogyrasinuosa* (Dana, 1846) (Scleractinia, Plerogyridae): characterisation and phylogenetic analysis

**DOI:** 10.3897/zookeys.1114.85028

**Published:** 2022-07-20

**Authors:** Peng Tian, Zhiyu Jia, Bingbing Cao, Wei Wang, Jiaguang Xiao, Wentao Niu

**Affiliations:** 1 Laboratory of Marine Biology and Ecology, Third Institute of Oceanography, Ministry of Natural Resources, Xiamen, China Third Institute of Oceanography, Ministry of Natural Resources Xiamen China

**Keywords:** Evolutionary relationships, mitogenome data, Plerogyridae, “Robust” clade

## Abstract

In this study, the whole mitochondrial genomes of *Physogyralichtensteini* and *Plerogyrasinuosa* have been sequenced for the first time. The length of their assembled mitogenome sequences were 17,286 bp and 17,586 bp, respectively, both including 13 protein-coding genes, two tRNAs, and two rRNAs. Their mitogenomes offered no distinct structure and their gene order were the same as other typical scleractinians. Based on 13 protein-coding genes, a maximum likelihood phylogenetic analysis showed that *Physogyralichtensteini and Plerogyrasinuosa* are clustered in the family Plerogyridae, which belongs to the “Robust” clade. The 13 tandem mitogenome PCG sequences used in this research can provide important molecular information to clarify the evolutionary relationships amongst stony corals, especially at the family level. On the other hand, more advanced markers and more species need to be used in the future to confirm the evolutionary relationships of all the scleractinians.

## ﻿Introduction

The order Scleractinia (Cnidaria, Anthozoa), including numerous reef-building coral species, is important as the constructors of coral reefs as an ecosystem. The mitogenome data of cnidarians contain important phylogenetic information for understanding their evolutionary history (Kayal et al. 2013). Single- or multiple-gene analysis of mitochondrial genes have already been used to infer phylogenetic relationships amongst scleractinians (Kitahara et al. 2016; Arrigoni et al. 2020).

In Scleractinia, three main clades have been defined based on molecular analyses, “Complex”, “Robust”, and “Basal” (Romano and Palumbi 1996; Kitahara et al. 2010; Stolarski et al. 2011). Plerogyridae Rowlett, 2020 is a small family of the “Robust” clade of corals containing four genera (*Plerogyra* Milne Edwards & Haime, 1848, *Physogyra* Quelch, 1884, *Blastomussa* Wells, 1968, and *Nemenzophyllia* Hodgson & Ross, 1982) (see Hoeksema and Cairns 2022), all from the Indo-West Pacific. Previously, the genera *Plerogyra* and *Physogyra* were placed in the Euphylliidae of the “Complex” group (Fukami et al. 2008), the family Plesiastreidae of the the “Robust” group (Dai and Horng 2009), and in Scleractinia incertae sedis (Budd et al. 2012; Benzoni et al. 2014; Waheed et al. 2015), but recently Rowlett (2020) placed them in the family Plerogyridae. *Physogyra* has one recently accepted species and four uncertain species, whereas *Plerogyra* has seven accepted species (Hoeksema and Cairns 2022). Through molecular analyses of two mitochondrial genes, Fukami et al. (2008) found that *Plerogyra* and *Physogyra* do not belong to the “Complex” clade of Scleractinia but to the “Robust” clade. Morphologically, plerogyrid species are characterised by mantle vesicles that are diurnally visible when the tentacles are partially retracted (Benzoni et al. 2014).

*Physogyralichtensteini* (Milne Edwards & Haime, 1851) and *Plerogyrasinuosa* (Dana, 1846) are covered by round to irregularly bifurcating vesicles during the day and active, open polyps at night (Veron 2000; Benzoni et al. 2014). *Physogyralichtensteini* is common in lagoons and reef slopes to deeper than 38 m (De Palmas et al. 2021). Colonies of *Physogyralichtensteini* are generally massive. They are meandroid, with short, widely separated valleys interconnected with a light, blistery coenosteum. Septa are large, solid, smooth-edged, exsert, and widely spaced. Walls are solid. Columellae are absent. Tentacles are extended only at night. The septal vesicles of *Physogyra* are considerably smaller and more numerous when compared to the closely related *Plerogyra*. The colour of *Physogyralichtensteini* is pale grey or sometimes dull green (Veron 2000), while in *Plerogyrasinuosa*, the colonies are flabello-meandroid with valleys somewhat connected by a light, blistery coenosteum. Living parts of colonies are sometimes separated by dead basal parts. Vesicles are the size of grapes and usually have the shape of grapes but may be tubular, bifurcated, or irregular, depending primarily upon the state of inflation. The colour of *Plerogyrasinuosa* is cream or bluish grey. *Plerogyrasinuosa* is a prominent species and reasonably common in protected reefs, and it is easily recognised by its bubbly appearance (Veron 2000; Rowlett 2020).

In this research, the complete mitochondrial genomes of *Physogyralichtensteini* and *Plerogyrasinuosa* are sequenced and their genome structures are analysed for the first time. The phylogenetic analyses of these two species, based on 13 protein coding genes (PCGs) of the mitogenome, in combination with another 42 species of other genera of Scleractinia and two species of Corallimorphidae Hertwig 1882 (order Corallimorpharia) as outgroups, because they are closely related to Scleractinia in evolutionary terms. This helps determine their phylogenetic status and facilitate further study on stony coral evolutionary and phylogenetic relationships.

## ﻿Materials and methods

### ﻿Sample collection and genomic DNA extraction

**Figure 1.**
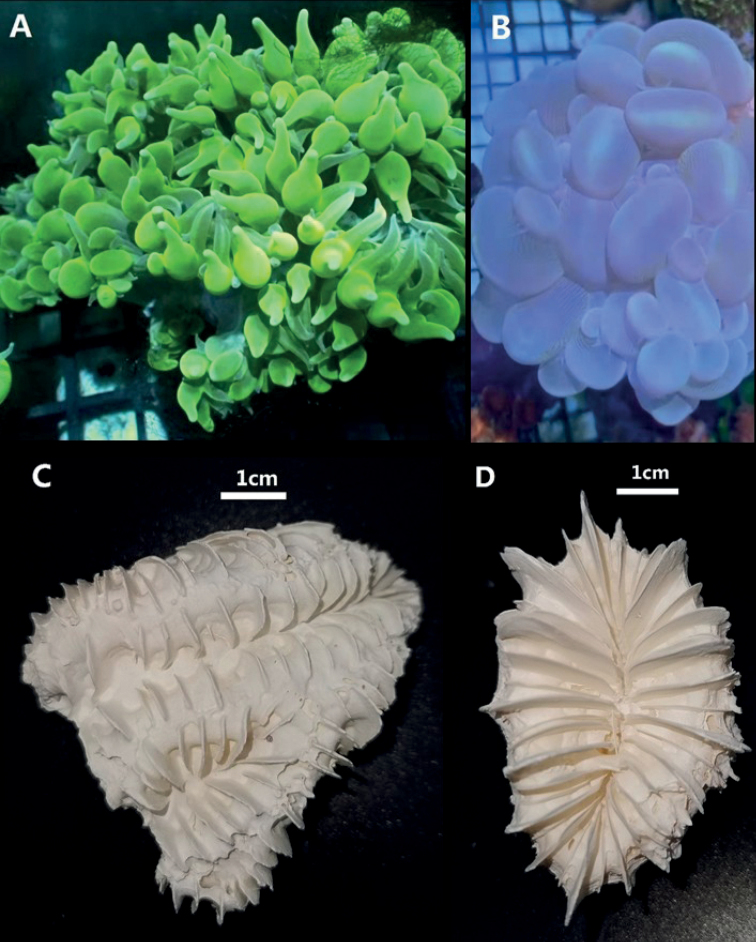
Scleractinian corals used in this study **A, C***Physogyralichtensteini***B, D***Plerogyrasinuosa***A, B** live animals **C, D** skeletons.

Samples of *Physogyralichtensteini* (Fig. 1A, C) and *Plerogyrasinuosa* (Fig. 1B, D) were obtained in 2019 from a coral mariculture company in China, which originally obtained mother stock from Negeri Sabah of Malaysia. Their specimens were maintained in our Coral Sample Repository with the codes 20191207-J2 (*Physogyralichtensteini*) and 20191207-Y1 (*Plerogyrasinuosa*). Total genomic DNA was extracted using the DNeasy Blood and Tissue Kit (Qiagen, Shanghai, China). Electrophoresis with 1% agarose gel was used to measure the integrity of their genomic DNA, and a NanoDrop 2000 spectrophotometer (Thermo Fisher Scientific, MA, USA) was used to measure their genomic DNA concentration.

### ﻿Mitogenome sequencing, annotation, and analyses

In this study, two methods were used to obtain the mitogenomes of *Physogyralichtensteini* and *Plerogyrasinuosa*, respectively. The complete mitogenome of *Plerogyrasinuosa* was obtained through a PCR approach using the primer pairs designed by Lin et al. (2011). The complete mitogenome of *Physogyralichtensteini* was obtained from high-throughput sequencing with a HiSeqX Ten platform (Illumina, San Diego, CA, USA) with a paired-end 150 bp approach according to Tian et al. (2021), and a total of 102,074 of 116,026,504 raw reads (approximately 0.09%) were de novo assembled to produce a single, circular form of the complete mitogenome with an average coverage of 892 X. The circularised contig of these two species were then submitted to the MITOS WebServer (Bernt et al. 2013; http://mitos.bioinf.uni-leipzig.de/index.py) for preliminary mitochondrial genome annotation. We identified and annotated their 13 PCGs and two rRNA genes by alignments of homologous mitogenomes from other scleractinians that had been recovered through BLAST searches in NCBI, and we also validated the tRNA genes using ARWEN (Laslett and Canbäck 2008). The genome structures were mapped using the online CGView Server (Stothard and Wishart 2005; https://proksee.ca/). Base composition, nucleotide frequencies, and codon usage were obtained through MEGA7 (Kumar et al. 2016). The skewing of the nucleotide composition was measured in terms of AT skews and GC skews according to the following formulae: AT skew = (A – T) / (A + T) and GC skew = (G – C) / (G + C) (Perna and Kocher 1995). The mitogenome sequences of *Physogyralichtensteini* and *Plerogyrasinuosa* are available in GenBank under accession numbers MW970409 and MW936598.

### ﻿Phylogenetic analyses

The phylogenetic positions of *Physogyralichtensteini* and *Plerogyrasinuosa* were inferred using 13 tandem mitogenome PCG sequences (ND5 + ND1 + Cytb + ND2 + ND6 + ATP6 + ND4 + COIII + COII + ND4L + ND3 + ATP8 + COI) (Tian et al. 2021) together with another 42 species of other genera of Scleractinia and two species belonging to two genera of Corallimorpharia that we obtained from GenBank (Table 1). We used MEGA 7 to select the best-fitting model based on the Akaike Information Criterion (AIC) and then constructed a maximum likelihood (ML) tree with 500 bootstrap replicates.

**Table 1.** Representative species of Scleractinia included in this study.

	**Species**	**Family**	**Mitogenome length (bp)**	**GenBank accession number**
1	*Physogyralichtensteini*	Plerogyridae	17,286	MW970409
2	*Plerogyrasinuosa*	Plerogyridae	17,586	MW936598
3	*Acroporahorrida*	Acroporidae	18,480	NC_022825
4	*Alveoporajaponica*	Acroporidae	18,144	MG851913
5	*Astreoporaexplanata*	Acroporidae	18,106	KJ634269
6	*Isoporapalifera*	Acroporidae	18,725	KJ634270
7	*Montiporacactus*	Acroporidae	17,887	NC_006902
8	*Agariciafragilis*	Agariciidae	18,667	KM051016
9	*Agariciahumilis*	Agariciidae	18,735	NC_008160
10	*Pavonaclavus*	Agariciidae	18,315	NC_008165
11	*Pavonadecussata*	Agariciidae	18,378	KP231535
12	*Desmophyllumpertusum*	Caryophylliidae	16,150	FR821799
13	*Solenosmiliavariabilis*	Caryophylliidae	15,968	KM609293
14	*Dendrophylliaarbuscula*	Dendrophylliidae	19,069	KR824937
15	*Tubastraeacoccinea*	Dendrophylliidae	19,094	KX024566
16	*Duncanopsammiapeltata*	Dendrophylliidae	18,966	NC_024671
17	*Fimbriaphylliaancora*	Euphylliidae	18,875	NC_015641
18	*Galaxeafascicularis*	Euphylliidae	18,751	NC_029696
19	*Colpophyllianatans*	Faviidae	16,906	NC_008162
20	*Mussaangulosa*	Faviidae	17,245	DQ643834
21	*Fungiacyathusstephanus*	Fungiacyathidae	19,381	JF825138
22	*Gardineriahawaiiensis*	Gardineriidae	19,430	MT376619
23	*Echinophylliaaspera*	Lobophylliidae	17,697	MG792550
24	*Dipsastraearotumana*	Merulinidae	16,466	MH119077
25	*Hydnophoraexesa*	Merulinidae	17,790	MH086217
26	*Orbicellafaveolata*	Merulinidae	16,138	AP008978
27	*Platygyracarnosa*	Merulinidae	16,463	JX911333
28	*Letepsammiaformosissima*	Micrabaciidae	19,048	MT705247
29	*Letepsammiasuperstes*	Micrabaciidae	19,073	MT706035
30	*Rhombopsammianiphada*	Micrabaciidae	19,542	MT706034
31	*Madreporaoculata*	Oculinidae	15,841	JX236041
32	*Plesiastreaversipora*	Plesiastreidae	15,320	MH025639
33	*Pocilloporaeydouxi*	Pocilloporidae	17,422	EF526303
34	*Seriatoporahystrix*	Pocilloporidae	17,059	EF633600.2
35	*Madracismirabilis*	Pocilloporidae	16,951	NC_011160
36	*Stylophorapistillata*	Pocilloporidae	17,177	NC_011162
37	*Gonioporacolumna*	Poritidae	18,766	JF825141
38	*Poritesfontanesii*	Poritidae	18,658	NC_037434
39	*Poriteslobata*	Poritidae	18,647	KU572435
40	*Poritesrus*	Poritidae	18,647	NC_027526
41	*Psammocoraprofundacella*	Psammocoridae	16,274	MT576637
42	*Astrangiapoculata*	Astrangiidae	14,853	NC_008161
43	*Pseudosiderastreatayami*	Siderastreidae	19,475	KP260633
44	*Siderastrearadians*	Siderastreidae	19,387	NC_008167
45	*Corallimorphusprofundus*	Corallimorphidae	20,488	KP938440
46	*Corynactiscalifornica*	Corallimorphidae	20,715	NC_027102

## ﻿Results and discussion

### ﻿Characteristics and composition of mitogenome

**Figure 2.**
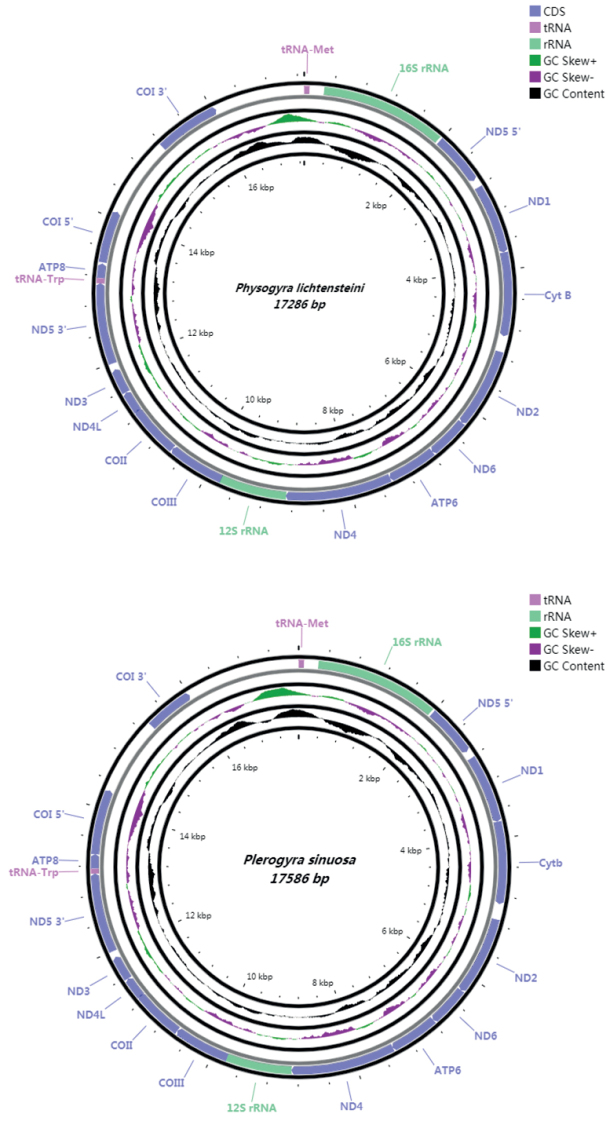
The mitogenome order and positions of *Physogyralichtensteini* and *Plerogyrasinuosa*. COI, COII, and COIII refer to the cytochrome oxidase subunits, Cyt *b* refers to cytochrome b, and ND1–ND6 refers to NADH dehydrogenase components. All the genes are encoded on the H-strand.

**Figure 3.**
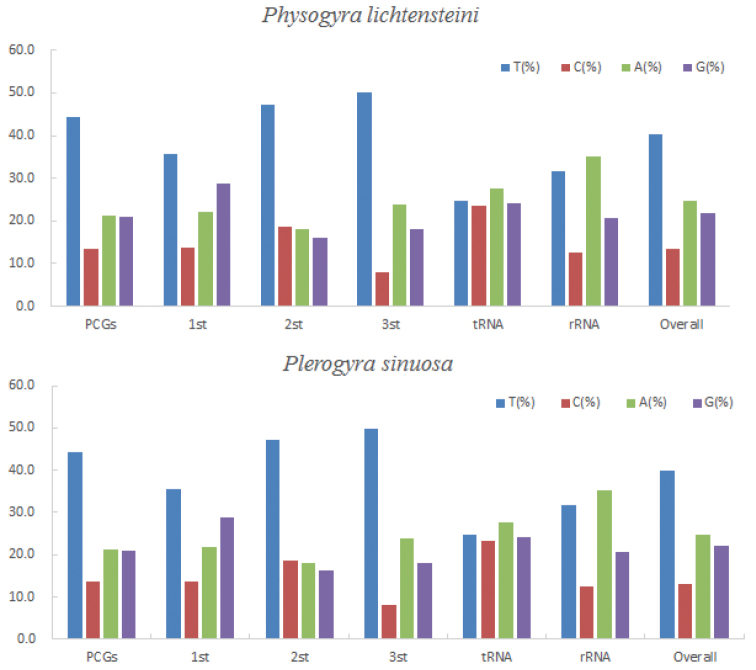
Codon usage bias in the different regions of the mitogenomes of *Physogyralichtensteini* and *Plerogyrasinuosa*.

The mitochondrial genome sizes of *Physogyralichtensteini* and *Plerogyrasinuosa* are 17,286 bp and 17,586 bp, respectively, both including 13 PCGs, two tRNA (tRNA^Met^, tRNA^Trp^), and two rRNA genes (Tables 2, 3; Fig. 2). Their mitogenomes offer no distinct structure and their gene orders are same as other typical scleractinians (Lin et al. 2012). All genes are encoded on the H-strand. The base composition of the complete mitogenome is 24.75% A, 13.32% C, 21.75% G, and 40.17% T for *Physogyralichtensteini*, and 24.87% A, 13.16% C, 22.01% G, and 39.96% T for *Plerogyrasinuosa.* Both species show a higher AT content than GC content (Fig. 3; Table 4).

**Table 2.** Organisation of the mitochondrial genome of *Physogyralichtensteini*.

Gene	Position		Length (bp)	Anticodon	Codon		Intergenic nucleotides*	Strand†
	From	To			Start	Stop		
tRNA^Met^	1	72	72	CAU			1228	H
16S rRNA	270	1967	1698				197	H
ND5 5’	1998	2708	711		ATG		30	H
ND1	2817	3764	948		ATG	TAG	108	H
Cytb	3767	4906	1140		ATG	TAA	2	H
ND2	5124	6218	1095		TTA	TAA	217	H
ND6	6219	6779	561		ATG	TAA	0	H
ATP6	6779	7453	675		ATG	TAA	−1	H
ND4	7453	8892	1440		ATG	TAG	−1	H
12S rRNA	8890	9800	911				−3	H
COIII	9794	10573	780		ATG	TAA	−7	H
COII	10576	11283	708		ATG	TAG	2	H
ND4L	11265	11564	300		ATG	TAG	−19	H
ND3	11567	11908	342		GTG	TAA	2	H
ND5 3’	11996	13099	1104			TAG	87	H
tRNA^Trp^	13098	13166	69	UCA			−2	H
ATP8	13170	13367	198		ATG	TAA	3	H
COI 5’	13385	14095	711		ATT		17	H
COI 3’	15173	16057	885			TAG	1077	H

*Data are number of nucleotides between the given gene and its previous gene; negative numbers indicate overlapping nucleotides. †H indicates that the genes transcribed on the heavy strand.

**Table 3.** Organisation of the mitochondrial genome of *Plerogyrasinuosa*.

Gene	Position		Length (bp)	Anticodon	Codon		Intergenic nucleotides*	Strand†
	From	To			Start	Stop		
tRNA^Met^	1	72	72	CAU			1581	H
16S rRNA	272	1969	1698				199	H
ND5 5’	2000	2710	711		ATG		30	H
ND1	2819	3766	948		ATG	TAG	108	H
Cytb	3769	4908	1140		ATG	TAA	2	H
ND2	5125	6219	1095		TTA	TAA	216	H
ND6	6220	6780	561		ATG	TAA	0	H
ATP6	6780	7454	675		ATG	TAA	−1	H
ND4	7451	8893	1443		ATG	TAG	−4	H
12S rRNA	8891	9797	907				−3	H
COIII	9795	10574	780		ATG	TAA	−3	H
COII	10577	11284	708		ATG	TAG	2	H
ND4L	11266	11565	300		ATG	TAG	−19	H
ND3	11568	11909	342		GTG	TAA	2	H
ND5 3’	11997	13100	1104			TAG	87	H
tRNA^Trp^	13099	13167	69	UCA			−2	H
ATP8	13171	13368	198		ATG	TAA	3	H
COI 5’	13368	14270	903		ATG		−1	H
COI 3’	15336	16004	669			TAA	1065	H

*Data are number of nucleotides between the given gene and its previous gene; negative numbers indicate overlapping nucleotides. †H indicates that the genes transcribed on the heavy strand.

**Table 4.** Nucleotide composition in different regions of mitogenomes of *Physogyralichtensteini* (*P.l.*) and *Plerogyrasinuosa* (*P.s.*).

Gene/Region	T (%)		C (%)		A (%)		G (%)		A+T (%)		Size (bp)	
	*P.l.*	*P.s.*	*P.l.*	*P.s.*	*P.l.*	*P.s.*	*P.l.*	*P.s.*	*P.l.*	*P.s.*	*P.l.*	*P.s.*
ND5	46.56	46.61	12.07	12.01	21.71	21.76	19.67	19.61	68.27	68.37	1815	1815
ND1	43.35	43.46	14.14	14.03	19.20	19.09	23.31	23.42	62.55	62.55	948	948
Cytb	44.91	44.82	13.68	13.68	20.88	20.88	20.53	20.61	65.79	65.70	1140	1140
ND2	47.31	47.31	12.79	12.60	20.00	20.09	19.91	20.00	67.31	67.40	1095	1095
ND6	44.56	44.56	13.37	13.55	22.28	22.28	19.79	19.61	66.84	66.84	561	561
ATP6	46.81	46.37	13.19	13.33	22.22	22.22	17.78	18.07	69.03	68.59	675	675
ND4	45.35	45.56	13.54	13.47	19.86	19.86	21.25	21.11	65.21	65.42	1440	1443
COIII	42.69	42.69	15.38	15.51	19.74	19.62	22.18	22.18	62.43	62.31	780	780
COII	39.69	39.55	13.28	13.28	24.01	23.87	23.02	23.31	63.70	63.42	708	708
ND4L	44.33	44.33	12.00	12.00	24.67	24.67	19.00	19.00	69.00	69.00	300	300
ND3	47.08	46.78	9.94	9.94	17.84	17.84	25.15	25.44	64.92	64.62	342	342
ATP8	43.43	43.43	12.12	12.12	29.29	29.29	15.15	15.15	72.72	72.72	198	198
COI	40.41	39.31	15.10	15.78	22.12	22.20	22.37	22.71	62.53	61.51	1596	1572
PCGs	44.35	44.20	13.42	13.50	21.25	21.25	20.98	21.06	65.60	65.45	11598	11574
1^st^	35.59	35.56	13.63	13.69	21.99	21.90	28.79	28.85	57.58	57.47	3866	3858
2^nd^	47.31	47.15	18.65	18.61	17.93	18.01	16.11	16.23	65.24	65.16	3866	3858
3^rd^	50.16	49.90	7.99	8.19	23.82	23.82	18.03	18.09	73.98	73.72	3866	3858
tRNA	24.82	24.82	23.40	23.40	27.66	27.66	24.11	24.11	52.48	52.48	141	141
rRNA	31.70	31.67	12.50	12.48	35.19	35.12	20.62	20.73	66.89	66.79	2609	2605
Overall	40.17	39.96	13.32	13.16	24.75	24.87	21.75	22.01	64.92	64.83	17286	17586

### ﻿Protein-coding genes

**Figure 4.**
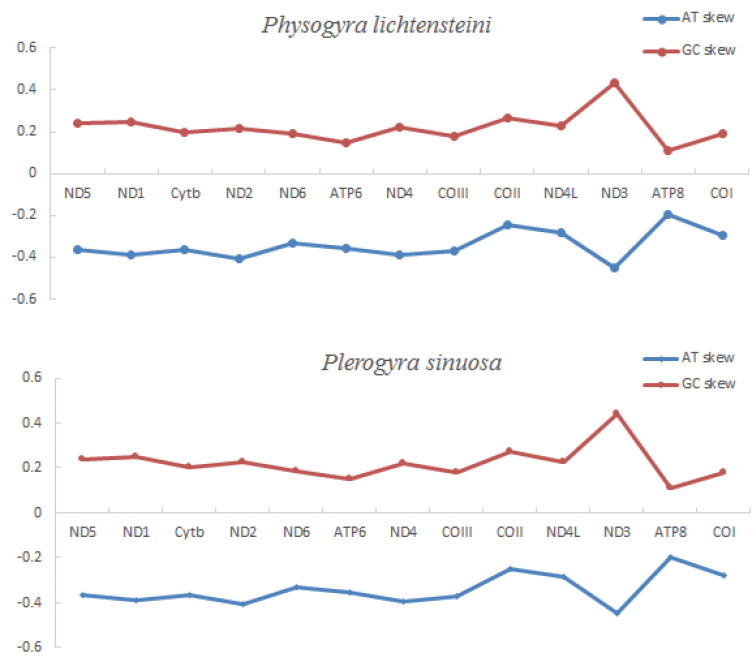
The PCG AT skew and GC skew of the mitochondrial genomes of *Physogyralichtensteini* and *Plerogyrasinuosa*.

**Figure 5.**
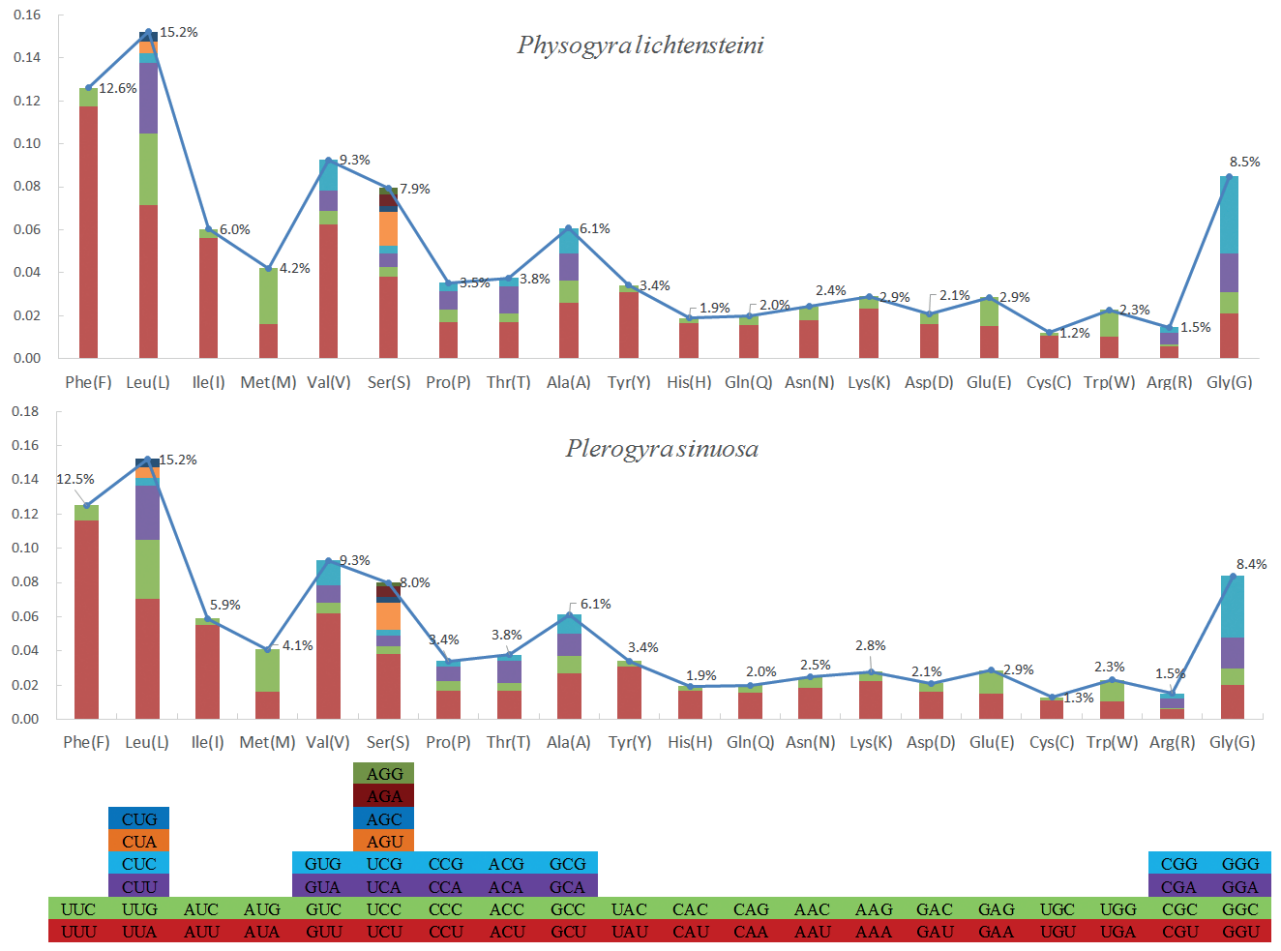
The PCG codon use frequency of the mitochondrial genomes of *Physogyralichtensteini* and *Plerogyrasinuosa*.

The lengths of all 13 protein-coding genes (PCGs) are 11,598 bp and 11,574 bp for *Physogyralichtensteini* and *Plerogyrasinuosa*, respectively. In both species, the ND5 gene and COI gene have intron insertions, and the start and stop codon of all 13 PCGs are exactly the same except for the COI gene. Their shortest gene is in both ATP8, and their longest gene is ND5 (Tables 2, 3). According to the AT-skew and GC-skew analyses (Fig. 4), both PCGs of *Physogyralichtensteini* and *Plerogyrasinuosa* show a stronger nucleotide asymmetry, with AT skew higher than GC skew. Amongst L, F, V, G, and S in *Physogyralichtensteini* and *Plerogyrasinuosa*, codon use frequency was higher, accounting for 53.5% and 53.4%, respectively. In their 20 amino acids, the majority are non-polar amino acids, and a minority are polarity-charged amino acids (Fig. 5).

### ﻿rRNA and tRNA genes

**Figure 6.**
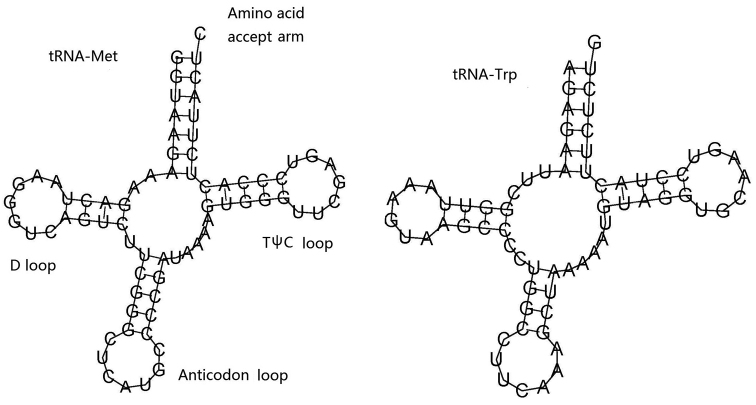
Putative secondary structures of two tRNAs in *Physogyralichtensteini* and *Plerogyrasinuosa*.

The encoding genes 12S and 16S rRNA in *Physogyralichtensteini* are 911 bp and 1,698 bp in size, and in *Plerogyrasinuosa* they are 907 bp and 1,698 bp in size. The base composition of rRNA in *Physogyralichtensteini* was 35.19% A, 12.5% C, 20.62% G, and 31.7% T, and in *Plerogyrasinuosa* it was 35.12% A, 12.48% C, 20.73% G, and 31.67% T. The two tRNA encoding genes, tRNA^Met^ (72 bp) and tRNA^Trp^ (69 bp), are exactly the same in *Physogyralichtensteini* and *Plerogyrasinuosa* (Tables 2, 3). They are folded into the classic cloverleaf structure which includes an amino acid accept arm, DHU loop, anticodon loop, and TψC loop (Fig. 6).

### ﻿Phylogenetic analyses

**Figure 7.**
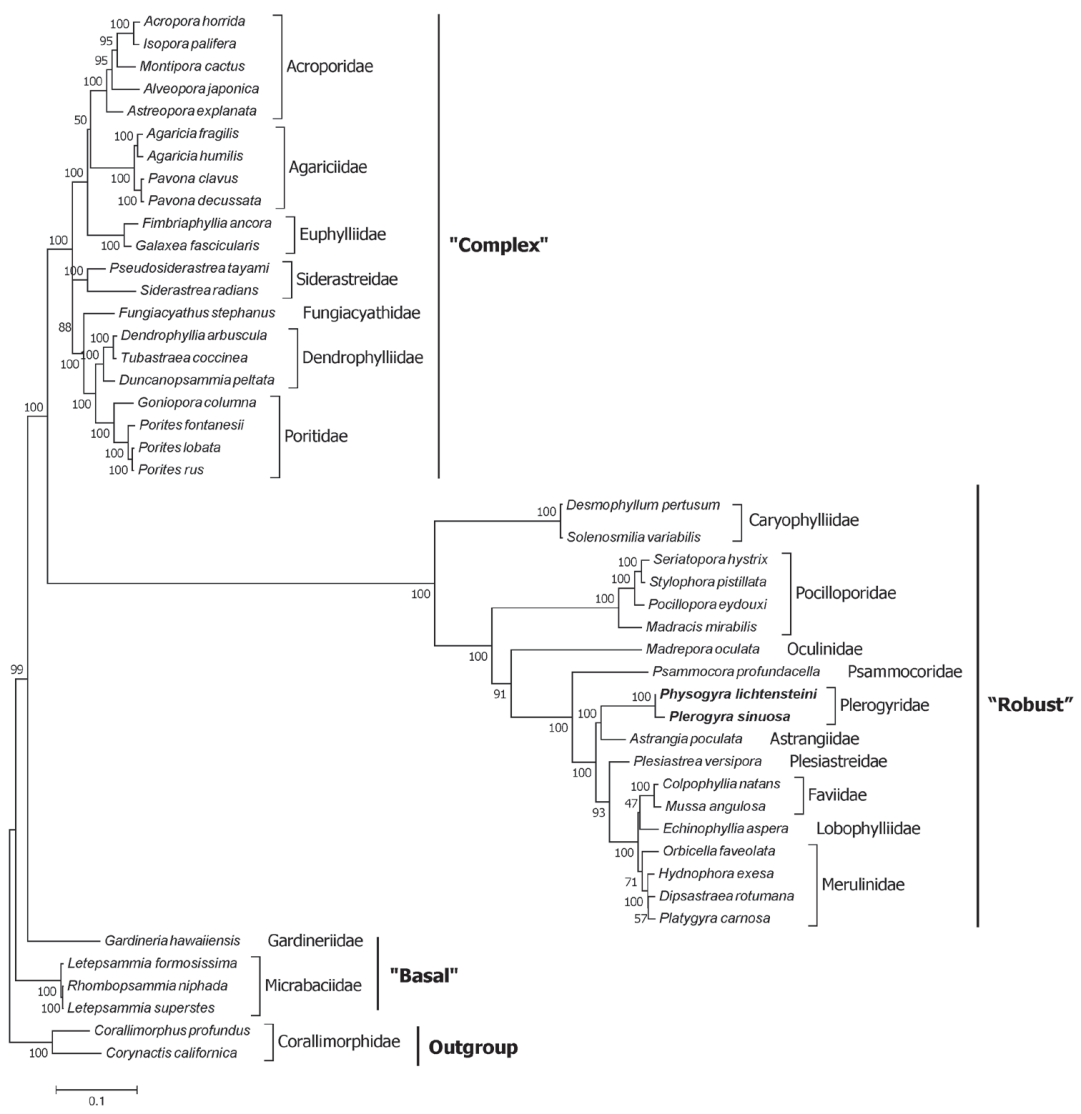
Inferred phylogenetic relationships based on a maximum-likelihood analysis of concatenated nucleotide sequences of 13 mitochondrial PCGs. Numbers on branches are bootstrap percentages.

There are three distinct clades of Scleractinia in our ML tree, including “Complex”, “Robust”, and “Basal” clade. The ML topology tree of all the 47 species shows that *Physogyralichtensteini* and *Plerogyrasinuosa* are clustered in family Plerogyridae which belong to the “Robust” clade with high bootstrap support (Fig. 7). Our finding is consistent with the results of Fukami et al. (2008) who placed *Plerogyra* and *Physogyra* in the “Robust” clade. From the ML tree we also find that *Physogyralichtensteini* and *Plerogyrasinuosa* are a sister group with *Astrangiapoculata*, which belongs to the family Astrangiidae Milne Edwards & Haime, 1857. Our MT tree of the Plerogyridae shows the same classification as used by Rowlett (2020). Single- or multi-gene analyses of mitochondrial genes have already been used to infer phylogenetic relationships amongst scleractinians (Kitahara et al. 2016; Arrigoni et al. 2020). The 13 tandem mitogenome PCG sequences we used in this research can provide important molecular information to understand the evolutionary relationships amongst stony corals, especially at the family level. As fewer than a tenth of stony coral species have been sequenced at this time, more mitogenomes of other scleractinians are necessary before accurate family-level evolutionary relationships can be reconstructed. In the future, more advanced markers and more species should be used to confirm the evolutionary relationships among all scleractinians.

## ﻿Conclusions

The complete mitochondrial genomes of *Physogyralichtensteini* and *Plerogyrasinuosa* were sequenced for the first time. Their mitogenomes show a similar gene order and composition with other typical Scleractinia. Our phylogenetic analysis of *Physogyralichtensteini* and *Plerogyrasinuosa*, based on their 13 tandem mitochondrial protein-coding genes and including another 42 species of Scleractinia and two species of Corallimorpharia, help us to understand the evolutionary relationships amongst stony corals and facilitate further studies on stony coral evolutionary and phylogenetic relationships.
